# Verbal Working Memory Processes in Students With Mild and Borderline Intellectual Disabilities: Differential Developmental Trajectories for Rehearsal and Redintegration

**DOI:** 10.3389/fpsyg.2018.02581

**Published:** 2019-01-09

**Authors:** Gunnar Bruns, Birgit Ehl, Michael Grosche

**Affiliations:** Rehabilitation Sciences on Special Learning Needs, Institute of Educational Research, School of Education, University of Wuppertal, Wuppertal, Germany

**Keywords:** working memory, mild intellectual disability (MID), borderline intellectual disability, rehearsal, redintegration, developmental trajectories, delayed onset, phonological loop

## Abstract

In verbal working memory, two processes serve to retain a fading memory trace: subvocal rehearsal and lexical redintegration. While recent studies on students with mild and borderline intellectual disabilities (MBID) have yielded mixed results on rehearsal, redintegration has not been researched in MBID, yet. Furthermore, most studies have used a group-matched design which, due to methodological constraints, can only distinguish between two different development patterns. Thus, we study both rehearsal and redintegration in students with MBID using developmental trajectories that have greater potential for identifying differential developmental patterns than traditional group-matching approaches. We investigate whether three aspects in working memory develop differently in students with MBID in comparison to typically developing students: (a) the general capacity of the phonological loop, and the effectiveness of (b) rehearsal, and (c) redintegration. We use three different developmental indicators to compare trajectories: chronological age, cognitive capacity, and vocabulary size. *N* = 210 students (87 students with MBID, 123 typically developing students) completed working memory span tasks with short and long (1- vs. 3-syllable) real words and pseudowords. The effect for word length (short vs. long) measures rehearsal, and the lexicality effect (real words vs. pseudowords) measures redintegration. Results show that developmental trajectories reveal an intercept difference but no slowed rate in rehearsal, and no impairment in redintegration. However, concerning the developmental relation between redintegration and vocabulary size, students with MBID reveal a differential pattern as redintegration appears higher for students with small vocabulary size, but unexpectedly decreases as vocabulary size increases. We conclude that students with MBID show a delayed onset in the development of capacity of the phonological loop and rehearsal and that they do not catch up in their development. Redintegration does not seem to be impaired in relation to age and cognitive capacity. However, the differential relation of redintegration with vocabulary size calls for further research. While impaired subvocal rehearsal appears to be connected to the developmental problems of students with MBID, lexical redintegration seems to be intact in relation to chronological age and cognitive capacity, making it a possible area of strength.

## Introduction

### Purpose of the Study

Mild and borderline intellectual disabilities (MBID) are defined as deficits in intellectual and adaptive functioning with an IQ between 55–70 (mild) and 70–85 (borderline). Most students with MBID have prominent deficits in academic learning. The etiology of MBID (van der Molen et al., 2014) is still largely unknown, apart from clearly diagnosable syndromes like Down syndrome or Williams syndrome.

Existing MBID etiology models are often simply lists of unconnected causal factors, describing the phenomenon only at a vague level (e.g., Schröder, 2000; Kretschmann, 2007; Shaw, 2010 as cited in Hassiotis, 2015), and lacking empirical foundation. Specific, coherent, and comprehensive theories about MBID are lacking. While most theories include cognitive processes and agree that information processing is likely to be impaired, they do not further elaborate on the nature of cognitive processes or the severity of impairment. Without such knowledge, we can neither improve our theories about MBID nor develop effective interventions.

Research on cognitive processes in MBID has focused on verbal working memory (Henry and MacLean, 2002; Hasselhorn and Mähler, 2007) and executive functions (Danielsson et al., 2010). However, different verbal working memory processes have seldom been studied together in the same sample, making it difficult to disentangle process differences from sampling differences. Furthermore, group mean matching protocols that are insensitive to differential developmental patterns have been used. Therefore, in this paper we use a novel methodology called developmental trajectories to investigate two cognitive processes in the same sample regarding verbal working memory, namely the well-researched phonological rehearsal process and the less common lexical redintegration process. Thus, the paper's contribution is two-fold: (1) the functionality of redintegration is investigated in students with MBID for the first time; and (2) the methodological extension of developmental trajectories allows to reveal differential patterns of cognitive development.

Our focus on cognitive processes does not claim to causally explain why MBID originate in the first place; nor does it neglect the relevance of other factors such as socio-cultural deprivation or inadequate education or schooling. We believe that insights in the development of cognitive processes can increase our knowledge about MBID and have the potential to effectively change theoretical models of MBID and education or intervention.

### Working Memory Theories

In our study, we focus on the overall capacity of two retention processes within verbal *working memory* (WM), namely *rehearsal* and *redintegration*. An influential framework for WM was developed by Baddeley (1986, 2000, 2012) positing four components: (a) the central executive with an (attentional) regulatory function that uses two modality-specific sub-systems; (b) the phonological loop (PL) for verbal information; (c) the visuo-spatial sketchpad for visual information; (d) the episodic buffer for binding information from different modalities. For typically developing children (TD), various studies established positive correlations of WM with academic outcomes (e.g., Alloway and Alloway, 2010; Poloczek et al., 2012; Mähler and Schuchardt, 2016), vocabulary learning (e.g., Gathercole et al., 1997; Baddeley et al., 1998), or thinking patterns such as inferencing and classification (Craig and Lewandowsky, 2013). Thus, a well-functioning WM is seen as an important prerequisite for learning and retaining memory traces until they can be incorporated in long-term memory (LTM).

In Baddeley's conceptualization of verbal WM, the PL consists of a phonetic store and a subvocal rehearsal process for retention. The phonetic store holds only a limited amount of information, mainly restricted by time due to decay or interference. Thus, the memory trace in the phonetic store degrades already after a few seconds (1.5–2.0 s; Hasselhorn and Mähler, 2007). To maintain the trace beyond the short retention of the phonetic store, the process of rehearsal has been proposed as a repeated subvocal articulation (Baddeley et al., 1984; Hasselhorn et al., 2000), i.e., the memorandum is pronounced silently. The amount of information that can be rehearsed depends on the articulatory speed and on the automatic activation of rehearsal (Hasselhorn et al., 2000). The general capacity of the PL results from the interplay between phonetic store and subvocal rehearsal.

The capacity of the PL and the effectiveness of the retention processes in maintaining the degrading trace are assessed in span tasks (Hasselhorn and Mähler, 2007), in which participants are asked to repeat a sequence of words or digits presented in a fixed rhythm of one element every 1–1.5 s. The highest number of elements recalled in their correct position serves as indicator of span capacity. Contrasting the performance for varying conditions of the recall stimuli (e.g., word length or lexicality) provides an insight into the effectiveness of the retention processes.

The automatic activation of rehearsal is traditionally operationalized as word length effect in word span tasks (e.g., Mähler and Hasselhorn, 2003): Short words (e.g., one syllable) are more likely to be remembered (correctly) than long words (e.g., three syllables) in span tasks (Baddeley et al., 1975). The word length effect is explained in terms of the effectiveness of rehearsal: Without rehearsal, only the last few seconds could be remembered in each condition alike. Silently articulating the stimuli is more effective for short words, because more short words than long words can be subvocally articulated in the same amount of time. Findings confirming that word span performance correlates with articulatory speed (Hasselhorn et al., 2000) and that the word length effect disappears under concurrent articulation (Baddeley et al., 1984) underline the phonological basis of rehearsal. In summary, the word length effect constitutes a seminal finding that supports the concept of rehearsal as an effective means for retaining a degrading memory trace in the PL of WM (but see Campoy, 2008 and Jalbert et al., 2011b).

Although verbal WM is originally conceptualized as only phonologically based, it is not independent of LTM. For instance, lexical attributes of stimuli in word spans, which are stored in LTM, have measurable effects on span performance. These lexical factors include word-frequency (Hulme et al., 1997), wordlikeness in pseudowords (Gathercole, 1995), lexicality (Gathercole et al., 2001), concreteness (Walker and Hulme, 1999; Allen and Hulme, 2006), and neighborhood size (Jalbert et al., 2011b; Clarkson et al., 2017).

The original PL-model does not specify a mechanism to account for these LTM effects. Lexical redintegration has therefore been proposed as a process explicitly linking WM to LTM that can account for lexical effects of word span tasks (Lewandowsky and Farrell, 2000). Redintegration aids recall in WM by using lexical information available in LTM to reconstruct a partially degraded memory trace (Schweickert, 1993; Thorn and Frankish, 2005; Roodenrys and Miller, 2008; Thorn and Page, 2009).

Redintegration is investigated empirically through word span tasks that contrast stimuli of different lexical conditions such as real words vs. pseudowords (Gathercole et al., 2001; Turner et al., 2004). Items differ in how readily they can be redintegrated “as a result of their properties in long-term memory” (Roodenrys and Miller, 2008, p. 579). The differential impact of lexical interference on words vs. pseudowords can be interpreted as a marker for redintegration (Conlin and Gathercole, 2006). As real words exist in LTM, their knowledge can be used for reconstruction. This does not apply to pseudowords, meaning that retention depends more heavily on the phonological form (Thorn et al., 2009).

Schweickert's (1993) model of multinomial processing trees incorporates both rehearsal and redintegration processes, although the reference to rehearsal remains implicit. If a memory trace is completely intact, it can be recalled directly. If, however, the trace is partially degraded, attempting to reproduce the recall series will lead to errors (Thorn et al., 2005). Via redintegration, the sequence can be reconstructed from the remainders of the degrading trace, based on pre-existing knowledge stored in LTM (Schweickert, 1993).

To conclude, the two WM processes of rehearsal and redintegration aid children in retaining memory traces that would otherwise quickly fade. It can hence be seen as a prerequisite for establishing stable LTM traces necessary for various academic tasks, e.g., learning new words where an unknown (phonological) word form has to be remembered, reading an instruction to understand the steps to solve a problem, or maintaining a sequence of steps during problem-solving.

### Working Memory Processes in Students With Mild and Borderline Intellectual Disabilities

To sum up, rehearsal and redintegration processes both play an important role in verbal working memory and thus in academic learning. The question is, though, how students with MBID use rehearsal and redintegration processes. Empirical findings regarding the PL and rehearsal in students with MBID are still inconclusive.

On the one hand, Hasselhorn and Mähler (2007) found a delayed development in the capacity of the PL for students with MBID that was in line with their slowed general intellectual development. Also regarding the effectiveness and automatic activation of the rehearsal process, there is evidence for a delayed development in accordance with the general intellectual development in children with mild intellectual disabilities (van der Molen et al., 2007), in adolescents with MBID (Mähler and Hasselhorn, 2003), and also when using non-verbal recall (Poloczek et al., 2016).

On the other hand, there is evidence for a more severe deficit in the PL domain that goes beyond the general poor mental development (van der Molen et al., 2007; Schuchardt et al., 2010, 2011). In adolescents with MBID, the subcomponent of the phonetic store seemed to be specifically impaired (Mähler and Hasselhorn, 2003). Regarding rehearsal, Hasselhorn and Mähler (2007) found a specific deficit in students with MBID, and Rosenquist et al. (2003) in students with MID aged 12–16 years. Other studies suggest that PL deficits seem to be connected to the severity of the intellectual disability (Henry, 2001) and the general mental development (Mähler, 2007).

Lexical redintegration has not been investigated in the population of students with MBID to date. For students with difficulties in reading and writing, Hasselhorn et al. (2010) found a trend for stronger redintegration, while children with dyscalculia showed stronger rehearsal. Grube et al. (2008) attributed differences in the phonological similarity effect to redintegration in typically developing children of 5 and 9 years. Marton and Eichorn (2014) investigated interactions between WM and LTM (without referring to the construct of redintegration) in children with specific language impairment. Henry (2010) found the interaction between LTM and WM in students with mild to moderate intellectual disabilities (IQ range 39–70) to be in line with their general development.

To conclude, we still do not know whether rehearsal and redintegration are equally available to students with MBID. Therefore, we examine these two processes in the present study to gain a better understanding of their role for students with MBID. If cognitive processes in these students turn out to be intact, they can be excluded as a causal factor in developmental models of MBID. If, on the other hand, we find these processes to be impaired, they must be considered as possible explanatory factors. It could help understand why students with MBID are facing academic challenges, and offer possible indication of training and intervention to help these students cope with limited working memory processes.

### Approaches to Assess Developmental Patterns

Research on developmental disabilities seeks to identify differential patterns of development in relation to typically developing control groups, e.g., in the “Developmental-Difference-Controversy” (Zigler and Balla, 1982). A group of students with MBID is matched with two control groups of typically developing students. While the first control group is matched for chronological age (CA), the second control group is matched for mental age (MA), i.e., raw scores in an intelligence test. This results in two scenarios: If the MBID group performs more poorly than the CA group but equally well as the MA group (MBID = MA < CA), the deficit in the target task is in line with their general lower cognitive performance, and a developmental *delay* is inferred. If the MBID group performs even worse than the MA group (MBID < MA < CA), this reveals a form of developmental *deviance*, as the performance in the target task is more affected than expected on the grounds of the general cognitive level. Thus, developmental delay can be excluded and a deviance in development is inferred.

Although this approach has driven a number of studies with important findings on the pattern of developmental disabilities, it has several shortcomings. These are described in more detail in Thomas et al. (2009), who therefore suggest *developmental trajectories* (DTs) as an alternative and more sophisticated approach of analysis. In brief, DTs aim “to construct a function *linking performance with age* on a specific experimental task and then to assess whether this function differs between the typically developing group and the disorder group” (Thomas et al., 2009, p. 336, italics added) and thus take variability within the groups into account. This procedure allows to fit a regression model for each group and task, and then to compare intercept and slope coefficients between the groups, tasks and their interactions.

While it is possible to include non-linear functions and zero-trajectories, the resulting three linear scenarios used for this study are: (a) *delayed onset*, which can be observed when the groups differ at the intercept, i.e., the onset of development; (b) *slowed rate*, which manifests as a difference in the slopes; and (c) a *combination* of delayed onset and slowed rate. Thomas et al. (2009) demonstrate that the traditional group matching approach cannot distinguish between the first two patterns. Furthermore, DTs allow to flexibly include different indicators of mental age (which we call *developmental indicators* [DI] in our study) to assess differential developmental relations, which is not possible in the group-matching approach either. Thus, the approach of DTs provides a richer taxonomy to describe how developmental pathways can be impaired (Thomas et al., 2009), which has been used in several studies to examine various pathologies (Annaz et al., 2008; Lei et al., 2011; Carney et al., 2013), but not for students with MBID yet. It should be noted that, following Thomas et al. (2009), this paper understands the term “development” as correlations with indicators for (mental) age, as data is cross-sectional and not longitudinal.

Given that all of the studies about MBID reported above have used a group-based matching approach, the mixed results might be at least partly due to methodological reasons. To better disentangle different developmental patterns of the PL capacity, rehearsal, and redintegration, the present study uses DTs as the methodology to establish whether students with MBID show differential patterns of verbal working memory development.

### Research Questions and Hypotheses

Does verbal working memory in students with MBID develop differently from typically developing students? We examine whether three different developmental indicators (chronological age, cognitive capacity, and vocabulary size) can predict the capacity of the PL and the effectiveness of rehearsal and redintegration processes in students with MBID compared with TD peers. Per definition, students with MBID reveal a reduced cognitive capacity, making it necessary to control for it. Vocabulary size is taken as an indicator of crystallized knowledge that is stored in LTM, allowing us to investigate the developmental relation between vocabulary size and redintegration which relies on the interaction between WM and LTM.

1. PL capacity: We expect to find either a delayed onset or a slowed rate in the development of the PL capacity in students with MBID because deficits that are in line with mental age have been shown in the literature. However, as noted above, these scenarios are not distinguishable in the traditional ANOVA approach.
2. Rehearsal: The effectiveness of rehearsal is also hypothesized to be impaired. However, as previous findings are mixed, no clear hypothesis can be built as to whether to expect a delayed onset or a slowed rate of rehearsal development, or both. Concerning the developmental relation with vocabulary size as a lexical variable, we expect no relationship with the phonologically based rehearsal process.
3. Redintegration: Regarding the effectiveness of redintegration, there are no studies on students with MBID from which theoretical assumptions could be derived. Since redintegration represents the use of information from LTM to infer items in the short-term store, it may be a source of cognitive impairment in students with MBID. If this is the case, a reduced redintegration effectiveness would be a differential finding that is (so far) unique to MBID. Here, we expect vocabulary size to relate to the redintegration process.

## Methods

### Participants

The sample included *N* = 210 German students: 87 belonged to the group of MBID (mild intellectual disability, IQ 55–70: 24 students; borderline intellectual functioning, IQ 70–85: 63 students), of whom 48 were male with a mean age of *M* = 12 years and 11 months, *SD* = 2.39, ranging from 7;4–17;1 years. One hundred and twenty-three students were in the typically developing (TD) group (51 male, *M* = 8 years and 5 months, *SD* = 1.87, range 6;0–13;5 years). IQ norm data were obtained from different sources: The IQ scores of students with MBID were supplied by the official assessment documents in the school records; the IQs of TD students were assessed using the Culture Fair Test family, depending on their ages (CFT 1-R, Weiß and Osterland, 2012; and CFT 20-R, Weiß, 2008, respectively). Detailed sample characteristics are described in Table 1. For raw score comparisons on cognitive capacity and vocabulary size, all students with MBID but only those TD students that fell into the sensitive range of the tests explained below were tested, resulting in a subsample of *n* = 102 TD students for mental-age comparisons.

**Table 1:** Sample characteristics and descriptive statistics: means and standard deviations of all variables for students with MBID and for TD students.

		**MBID (*****n*** **=** **87)**		**TD (*****n*** **=** **123)**		**Significance**
		**M**	**SD**	**M**	**SD**	
Sex (male/female)		48/39		51/72		$\chi_{\left(1\right)}^{2}$ = 3.31; *p* = 0.069; φ = 0.14
Age (years; months)		12;11	2;6	8;5	1;10	t_(155.74)_^a^ = 14.9; *p* < 0.001; *d* = 2.1
Cognitive Capacity (CFT 1-R raw scores)		63.7	13.2	65.4^b^	13.5	*t*_(180)_ = 0.85; *p* = 0.395; *d* = 0.13
Vocabulary size (WWT 6–10 raw scores)		53.1	15.9	54.7^b^	14.6	*t*_(180)_ = 0.70; *p* = 0.483; *d* = 0.10
Intelligence (IQ norm scores)		74.3	7.3	105.3	10.2	t_(207.98)_^a^ = 25.7; *p* < 0.001; *d* = 3.60
**WORD SPAN PERFORMANCE CONDITION**						
Word span Real words	Short	3.27	0.57	3.47	0.64	*t*_(208)_ = 2.43; *p* = 0.016; *d* = 0.35
	Long	2.71	0.44	2.79	0.55	*t*_(208)_ = 1.18; *p* = 0.240; *d* = 0.17
	Total real words	2.99	0.46	3.13	0.55	*t*_(208)_ = 2.03; *p* = 0.043; *d* = 0.29
Word span Pseudowords	Short	2.62	0.66	2.76	0.74	t_(204)_^c^ = 1.38; *p* = 0.169; *d* = 0.20
	Long	1.71	0.43	1.79	0.54	t_(203)_^c^ = 1.09; *p* = 0.277; *d* = 0.16
	Total pseudowords	2.16	0.51	2.28	0.59	t_(203)_^c^ = 1.44; *p* = 0.151; *d* = 0.21

*The Culture-Fair-Test 1 Revision (CFT 1-R; Weiß and Osterland, 2012) raw scores were used to assess cognitive capacity as developmental indicator (DI); the picture naming vocabulary test “Wortschatz- und Wortfindungstest für 6–10 Jährige” (WWT 6–10, Glück, 2011) raw scores were used to assess vocabulary size as DI; Intelligence norm values were obtained from different sources: for students with MBID from the official school records, for younger TD students the corresponding CFT 1-R was administered and norm values were calculated, and for older TD students (ten years and older) the CFT 20-R (Weiß, 2008) was administered and norm values were calculated*.

a *dfs are corrected due to unequal variances*.

b *Subsample of n = 102 students in the TD group to avoid ceiling effects*.

c *Drop-out of five students (2 with MBID, 3 TD) who did not complete the session with word span tasks with pseudowords*.

To qualify for the MBID group, students had to fulfill the following criteria: a formal diagnosis of special learning needs (in German “Sonderpädagogischer Förderbedarf im Lernen”); an IQ below 85 as measured during the formal special needs assessment; no other developmental disorders, such as ADHD, ASD, and specific learning disabilities (dyslexia, dyscalculia), according to teacher report. All students with MBID were sampled from four special educational needs schools (“Förderschule Förderschwerpunkt Lernen”) in an urban environment in Western Germany. These schools constitute a special institution for students who reveal severe and continuous difficulties in academic learning, leading to the diagnosis of “special educational needs in learning.” Class size typically does not exceed 15 students. Students of the TD group attended a mainstream school (primary school or secondary school), had no diagnosis of special educational needs or developmental disorders, and their IQ and vocabulary scores had to be at least average (i.e., IQ > 85; vocabulary T-Score > 40). Class size typically ranges between 20 and 28 students in primary and 28–31 students in secondary grades. Participants were excluded if they could not follow the instructions. Data regarding monolingual status were not obtained; however, none of the participating children revealed difficulties in understanding the instructions, according to the administrators' judgement.

The study was carried out in accordance with the recommendations of the German Psychological Society, i.e., that written informed consent be obtained from all subjects' parents or caregivers in accordance with the Declaration of Helsinki. All participants gave their oral agreement to participate, and their parents and/or legal guardians were informed of the objectives of the study, the nature of the tasks that would be administered, and the fact that they could withdraw their agreement at any time. Permission to conduct tests in schools was obtained from the school principals.

### Procedure and Materials

Each student was tested in a group session on cognitive capacity, and in two individual sessions on the experimental tasks lasting ~30 min each. For the TD group, the first session contained the WM tasks. The second session involved a picture-naming task on vocabulary size. For the MBID group, the structure was slightly rearranged to enhance compliance: the first session involved only one WM task and the second session one WM task and the vocabulary task. The TD students at secondary schools completed the vocabulary task in a group setting.

#### Word Span Tasks

We used four different word span tasks in 2 (Length: short vs. long) × 2 (Lexicality: real vs. pseudo) conditions (e.g., short real word span: “Haus–Stern–Schuh” [house, star, shoe], or long pseudo word span: “karflumen–franulich–wuralten”). For the real word condition, we used the words from the standardized German Working Memory Test Battery (AGTB 5-12, Hasselhorn et al., 2012), and for the pseudoword condition, we used the pseudowords from a study by Hasselhorn et al. (2010; see Table A1 in the Appendix). The construction of word span sequences in all four conditions followed the principles of the AGTB 5-12. Sequences varying from two to eight words in length were presented in a 1.5 s rhythm. Each sequence was randomly assembled from a pool of nine words, spoken by a female voice, considering that the order of words within a sequence did not resemble other sequences. For each sequence length (i.e., number of words per sequence), 11 different sequences with pseudorandomized word order were provided in a list of audio files on a PC, from which they were presented aurally. The number of items per sequence was adaptively chosen based on the child's previous performance (detailed procedure described below). The students were instructed to listen to the sequence until the final signal and to repeat the complete sequence as correctly as possible. This procedure was practiced in three trials before the actual task. While the order of lexicality was randomly assigned, the order of length conditions was fixed across all individuals: Short words were followed by long words. For the pseudowords, children were told that the words might sound unfamiliar “like in a secret language of aliens.”

The word span tasks were adaptive. In two calibration items at the beginning of each condition, sequence length was adapted after every item. If it was repeated correctly, length was increased by one word; if it was not correct, length was decreased by one. The minimum length of a sequence was two words. The following eight test items were used for calculating the word span score and adaption took place after every other item: If Sequences 3 and 4 were both correct, the next two sequences would be one word longer; if both were incorrect, the next two would be one word shorter; if one was correct and one incorrect, length remained at the current level. Correct repetitions were awarded points corresponding to the length of the sequence, an incorrect repetition received points equal to the sequence length minus 1 (e.g., incorrect repetition of a four-word sequence was awarded three points). The overall word span score per condition was computed as the mean of the points of the eight test items. If all sequences were repeated incorrectly, the total score would be “1”; the theoretical maximum would be “7.5” when all test items were repeated correctly. We computed the internal consistency of the four condition scores (short real words, long real words, short pseudowords, long pseudowords) to be *r* = 0.83 for TD children and *r* = 0.84 for students with MBID.

#### Cognitive Capacity

The CFT 1-R (Weiß and Osterland, 2012) was administered to all students with MBID and a subsample of younger students in the TD group (*n* = 102). The CFT 1-R is a language-free intelligence measure, consisting of six subtests: substitutions, labyrinths, similarities, continue sequences, classification, and matrices. Norms are provided for age 5;4–9;11, with retest reliability reported to be at *r*_tt_ = 0.90 and internal consistency at *r* = 0.97. As the raw score is used as a matching criterion to investigate developmental relations, norm scores are not necessary and therefore it is not problematic that the age of the MBID group exceeds the range of age for which the CFT 1-R is normed. Instead, it is more relevant that the raw scores fall within a sensitive range, i.e., that no ceiling or floor effects exist. In the primary schools, the CFT 1-R was administered as a group test of up to 15 students. The setting in the special schools (MBID group) was in single sessions or small group sessions (maximally 4 students). The group of older TD students (secondary school) was tested in a group session on the CFT 20-R (Weiß, 2008), which provides norms for their age range (8;5–60;0 years), to ensure that these students met the inclusion criterion (no intellectual disabilities). Retest-reliability is *r*_tt_ = 0.80 and internal consistency *r* = 0.95.

#### Vocabulary Size

Students with MBID and the younger TD students were tested at an individual session with a computer-based test on expressive vocabulary in German for primary students aged 6 to 10 years (*Wortschatz- und Wortfindungstest 6–10*, WWT 6–10, Glück, 2011). The test consists of 95 images representing (parts of) objects, activities, opposites, and categories. The child is prompted: “what is this; what is he/she doing; what is the opposite of…; what are these things altogether.” Each item is shown on the screen for maximally 15 s. Reliability estimates are reported as *r*_tt_ = 0.96. The older TD group (secondary school) completed the vocabulary test that is part of the CFT 20-R (Weiß, 2008) during the group session. For a given word, a synonym has to be marked from a choice of 5 options (e.g., target-word: “fantasy.” Options: “form,” “principle,” “illusion,” “imagination,” “apprehension”) over 30 items; reliability is reported in the manual to be *r*_tt_ = 0.87. Note that the latter test was only used for sample selection purposes, to ensure that older control students' vocabulary was in the normal range. The group comparisons on vocabulary size were solely carried out based on the expressive WWT 6–10 raw scores that were completed by a subsample of *n* = 102 TD students.

### Analyses

The analyses of developmental trajectories (DT) follow a series of steps of increasing complexity. The results are depicted in scatterplots with regression lines and confidence intervals. Regression analyses are carried out to test whether the effects are statistically significant (Thomas et al., 2009). In the following analyses, the word span score is always the dependent variable. In all analyses, we establish three kinds of predictors: (a) three continuous predictors for (mental) age, which we call *developmental indicators* (DIs); (b) one between-subjects predictor (*group factor*); and (c) two within-subjects predictors, called *task conditions*. In total, we construct nine developmental trajectories: one DT across groups for all three dependent variables for each of the three DIs.

The three DIs comprise chronological age (CA) and two mental age variables: cognitive capacity (COG) and vocabulary size (VOC). The DIs establish whether a measure of chronological or mental age can reliably predict performance in the word span tasks. The group factor differentiates between students with MBID and the TD group for two purposes: first, to test whether the groups differ in their overall word span scores, and second to investigate interactions with DIs and task conditions. Lastly, the task condition predictors encompass the 2 × 2 different word length (short vs. long) and lexicality (real vs. pseudo) conditions. Through a task effect for word length (short words are more likely to be recalled than long words), rehearsal can be measured; the task effect for lexicality (real words are more likely to be recalled than pseudowords) estimates redintegration.

The first step of constructing a DT is to fit a regression model with the word span performance as dependent variable and a DI as predictor. This indicates whether there is any reliable relationship between the DI and word span performance. In the second step, the group factor is added to compare the groups regarding their intercepts and slopes, which are estimated separately for each group. The main effect for group denotes whether a difference exists in the intercepts between the groups. The interaction Group × DI considers the difference in slopes to establish whether the groups differ in their correlation between DI and word span performance (i.e., whether students with MBID catch up or if the gap widens). As the third step, one or more task conditions can be included to determine whether intercepts and slopes differ for various types of tasks. Again, the main effect for task reveals whether there is a general advantage for e.g., short words over long words (word length effect) or real words over pseudowords (lexicality effect) at the intercept. The interaction Task × DI shows the difference in slopes indicating that the relationship between DI and word span performance differs for the conditions of the task (e.g., if the advantage for short words increases with age). As the fourth step, the full model with all three predictors (task, group, and DI) is analyzed to establish whether there are differential developmental patterns for different task conditions. The Task × Group interaction denotes the differences of intercepts, whether e.g., the word length effect (advantage of short words over long words) is different between the groups, and hence show whether students with MBID show a delayed onset of development. Finally, the triple interaction Task × Group × DI represents the difference in slopes indicating that the relationship between DI and task effects differs between the groups (e.g., whether the advantage for short words over long words increases with age in both groups equally). This effect denotes whether students with MBID show a slowed rate of development in comparison to the TD group.

We will compare the respective intercept and slope effects for the dependent variables capacity of the PL (Hypothesis 1), effectiveness of the rehearsal process (Hypothesis 2), and effectiveness of the redintegration process (Hypothesis 3). For each dependent variable, we report the developmental relations with all three DIs; of particular interest is the developmental relation between vocabulary size and redintegration (Hypothesis 3). To analyze the capacity of the PL, the dependent variable of word span scores is predicted by the three different DIs and the group factor, without accounting for the task conditions; thus, word span scores are averaged over all 2 × 2 task conditions. Rehearsal is operationalized by the word length effect. Therefore, the section on rehearsal takes word length as predictor into the model, and word span scores are averaged over both lexicality conditions. Analogously, lexicality is taken as predictor into the model to investigate redintegration. Here, word span scores are averaged over both length conditions.

For the computation of regression coefficients, the developmental indicators are linearly rescaled such that the minimum value of the group of children with MBID is located at 0. This only affects the size of the intercept coefficient to allow a meaningful interpretation of the intercept difference between groups (Thomas et al., 2009), because extrapolation beyond the range of the data is avoided. The slope effect is not affected by this transformation. Rescaling is carried out only for the purpose of the statistical calculation of intercept effects, while the original ages are still shown in the figures. The detailed regression models' statistics (*F, p*, η^2^) can be found in Table A2 in the Appendix; as for readability, only *p*-values are reported in the text.

Data were prepared and figures created in R (R Studio Team, 2016; R Core Team, 2018) using the packages ggplot2 (Wickham, 2011) and ggpubr (Kassambara, 2017). Regression analyses of DTs were performed in SPSS Version 25 (IBM Corp, 2017) following the procedure outlined in the electronic supplement in Thomas et al. (2009).

## Results

Sample characteristics and descriptive results of mean word span scores for short and long real- and pseudoword conditions are provided in Table 1. Correlations between the three DIs (chronological age, cognitive capacity, vocabulary size) can be seen in Table 2. The order of lexicality in the WM task was randomly chosen in a subsample yielding no significant effects whether real words were administered first or second, *t*_(118)_ = 0.20; *p* = 0.844. To test possible gender effects, two linear mixed models were estimated for each DI. A baseline model included all predictors (i.e., DI, group, length, and lexicality), and the full model additionally included the factor gender. Models were compared regarding their fit using a Chi-square statistic. None of the three models gave evidence for a significant gender effect: chronological age, $\chi_{\left(16\right)}^{2}$ = 14.525; *p* = 0.560; cognitive capacity, $\chi_{\left(16\right)}^{2}$ = 13.418; *p* = 0.642; and vocabulary size, $\chi_{\left(16\right)}^{2}$ = 12.642; *p* = 0.699. Thus, data were pooled across both gender groups.

**Table 2:** Correlations for developmental indicators for students with mild and borderline intellectual disabilities (MBID below the diagonal) and typically developing students (TD above the diagonal).

		**TD (*****n*** **=** **102)**		
		**Chronological age**	**Cognitive capacity**	**Vocabulary size**
MBID (*n* = 80)	Chronological age	–	0.74	0.73
	Cognitive capacity	0.42^a^	–	0.67
	Vocabulary size	0.45^a^	0.49^b^	–

*Pearson correlations between developmental indicators (all p < 0.001)*.

*Due to unsystematic missing data in the MBID group, sample size is smaller than the full sample of n = 87 children*.

a *n = 80*.

b *n = 73*.

### Capacity of the Phonological Loop

**Figure 1:**
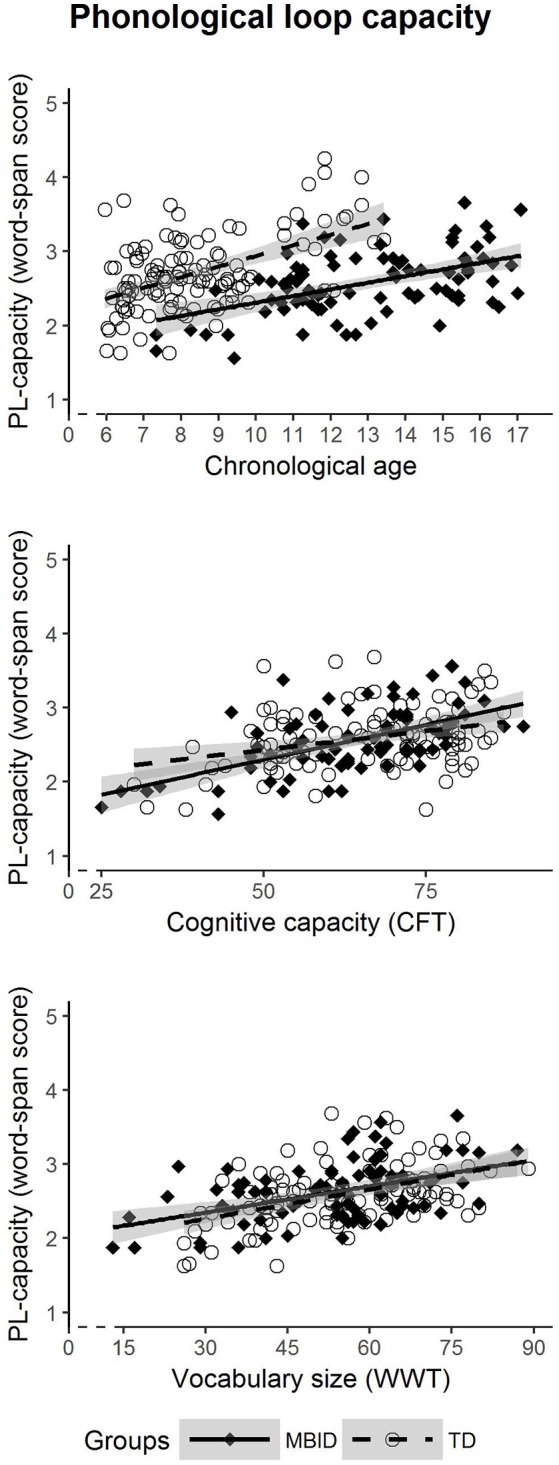
Developmental trajectories of phonological loop capacity in students with MBID (filled diamonds) and TD students (circle).

In the first analysis (Figure 1), we examined whether students with MBID show differential developmental patterns regarding the general capacity of the PL. This answers the question as to whether the development of PL capacity in students with MBID starts at the same onset level as in TD students; and we can determine if students with MBID tend to catch up with TD students on a possible PL capacity deficit with increasing age, cognitive capacity or vocabulary size; or if they continue to lag behind TD students. PL capacity was measured as the overall word span scores averaged over all 2 × 2 task conditions. Thus, only the group factor and the DIs were taken into the model as predictors, without distinguishing between different levels of length and lexicality. For each DI (chronological age, cognitive capacity, vocabulary size), we compared the groups concerning the intercepts (measured as group main effect) and the slopes (measured as Group × DI interaction).

When using *chronological age* as DI to predict PL capacity, we found a significant intercept effect for group (*p* < 0.001), as students with MBID had a lower intercept. The interaction of Group × Age, denoting a slowed rate of development, did not reach significance (*p* = 0.068). This means that students with MBID show a delayed onset in the development of PL capacity, but their rate of development in relation to chronological age was not significantly lower. With *cognitive capacity* as DI to predict PL capacity, we found a significant intercept difference for group (*p* = 0.039), and a significant interaction Group × COG (*p* = 0.033) showing a difference in slopes. The onset of development was significantly delayed and the slope for students with MBID was significantly steeper, indicating a stronger relationship between cognitive capacity and general PL capacity than in TD students. Thus, students with MBID reveal a delayed onset, in combination with a differential rate in PL capacity development in relation to their cognitive capacity. However, it should be noted that cognitive capacity scores may be confounded with chronological age; removing younger students with lower cognitive capacity scores (CFT 1-R raw scores < 45) from the analyses left both effects nonsignificant, i.e., *p* = 0.081 for the intercept difference and *p* = 0.073 for the difference in slopes. For *vocabulary size* as DI to predict PL capacity, neither the intercept difference for group was significant (*p* = 0.331) nor did the slopes differ (Group × VOC; *p* = 0.595). This indicates that the developmental pattern of PL capacity does not differ in relation to vocabulary size.

In summary, regarding the general capacity of the PL as measured by the word span scores across all task conditions, students with MBID revealed only a delayed onset of their PL but showed the same rate of development over the chronological age range. Cognitive capacity turned out to be a stronger predictor for PL capacity in students with MBID than in the TD group; this did not hold, however, when children with lower raw scores of cognitive capacity were removed from the analysis. No differential effects could be observed in terms of vocabulary size on PL capacity. Vocabulary size as DI does not produce any differential patterns between the MBID and TD groups.

### Effectiveness of the Rehearsal Process

**Figure 2:**
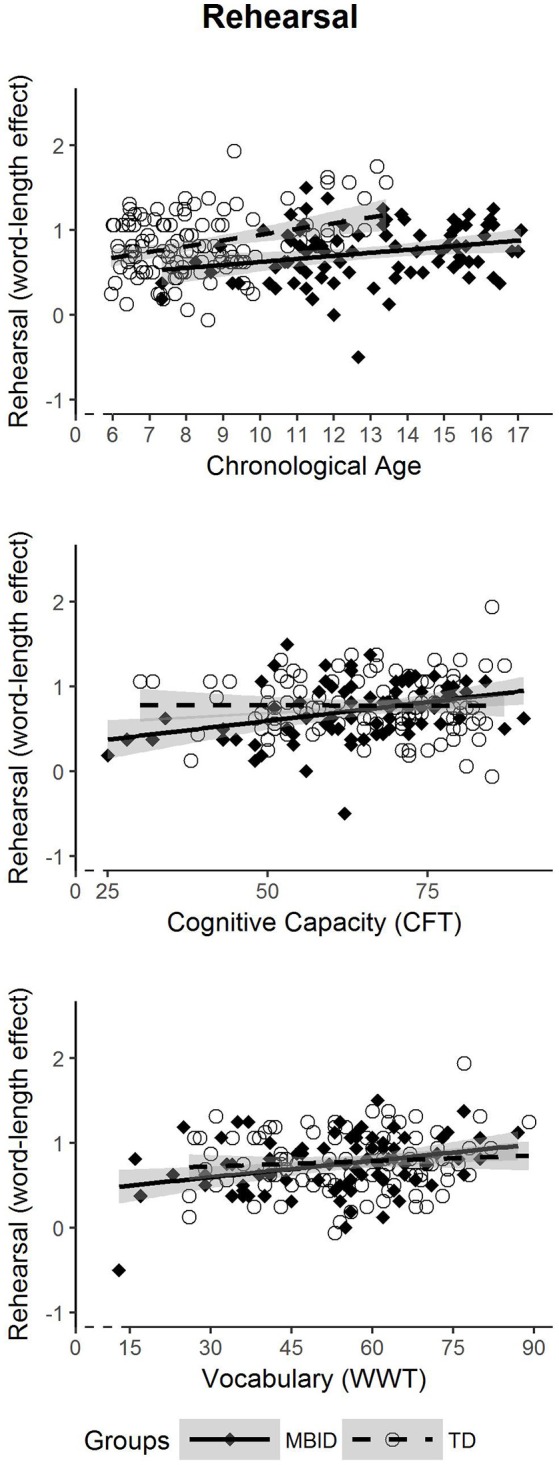
Developmental trajectories of rehearsal effectiveness in students with MBID (filled diamonds) and TD students (circle).

In the second analysis (Figure 2), we examined whether students with MBID show differential developmental patterns regarding the effectiveness of rehearsal. As in the first research question, this answers the question as to whether the effectiveness of rehearsal in students with MBID starts at the same onset level as in TD students; and we can determine whether developmental progress (in terms of age, cognitive capacity and vocabulary size) reflects in a similar way onto rehearsal development as in TD students. As rehearsal is operationalized as word length effect (i.e., a relative benefit for short words over long words), the task condition length is also included as predictor besides the group factor and the DIs, but without distinguishing between different levels of lexicality. For ease of interpretation, Figure 2 shows the size of the word length effect (the difference between short and long words) in the y-axis, whereas in the regression model, length is included as predictor.

Using *chronological age* as DI to predict rehearsal, a significant intercept effect for Length × Group (*p* = 0.021) was found, while the interaction of Length × Group × Age, denoting the rate of development, was not significant (*p* = 0.210). This means that students with MBID show a delayed onset, but no slowed rate in rehearsal development in relation to chronological age. With *cognitive capacity* as DI to predict rehearsal, the Length × Group interaction (*p* = 0.009) yielded a difference in intercept, and a significant interaction Length × Group × COG (*p* = 0.016) showed a difference in slopes. The slope for students with MBID was significantly steeper, indicating a stronger relationship between cognitive capacity and rehearsal effectiveness than in TD students. Thus, students with MBID reveal a delayed onset, in combination with a steeper rate in rehearsal development in relation to cognitive capacity. Again, removing younger students with lower cognitive capacity scores (raw scores < 45) from the analyses left both effects nonsignificant, i.e., *p* = 0.147 for the intercept difference and *p* = 0.167 for the difference in slopes. *Vocabulary size* as DI did not predict differences between the groups. Neither the intercept difference (Length × Group; *p* = 0.212) nor the slope difference (Length × Group × VOC; *p* = 0.192) became significant. This shows that the developmental pattern of rehearsal effectiveness does not differ in relation to vocabulary size.

To summarize, rehearsal development is delayed in onset for students with MBID when using chronological age as developmental indicator. A differential rate of rehearsal development in students with MBID when accounting for cognitive capacity as DI should be interpreted with caution, as removing children with lower COG scores removed the intercept and slope differences, suggesting that the relationship between rehearsal and cognitive capacity is similar in both groups. In neither group did lexical knowledge (vocabulary size) have any significant predictive power.

### Effectiveness of the Redintegration Process

**Figure 3:**
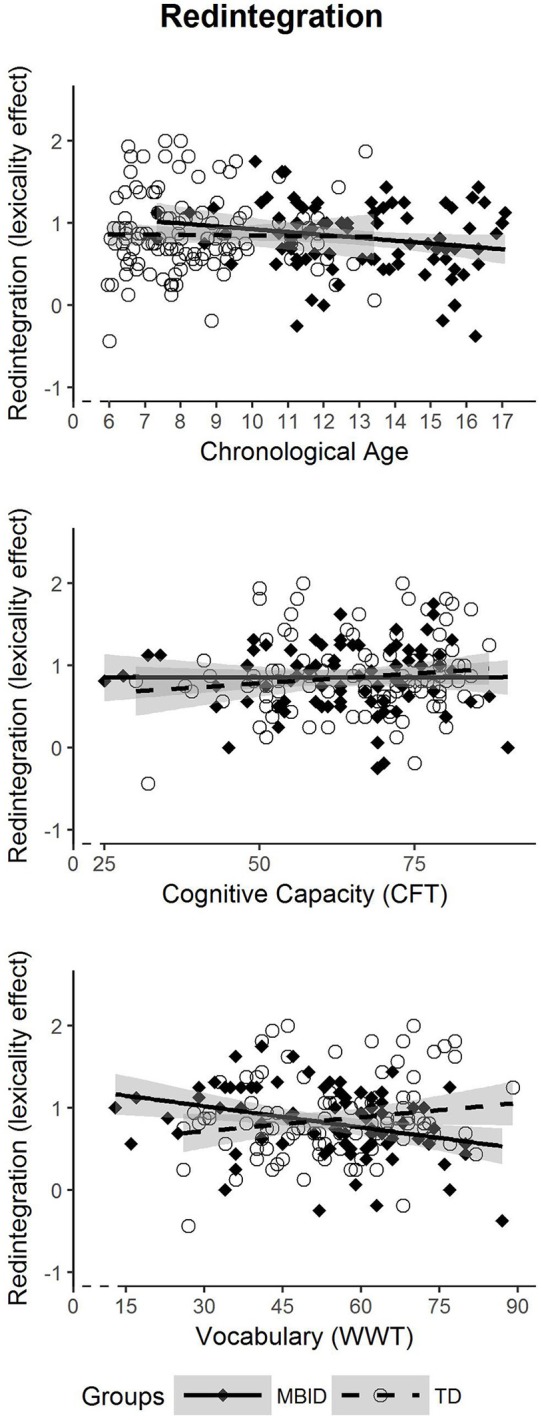
Developmental trajectories of redintegration effectiveness in students with MBID (filled diamonds) and TD students (circle).

In the third analysis (Figure 3), we examined whether students with MBID show differential developmental patterns regarding the effectiveness of redintegration. As before, this answers the question as to whether the effectiveness of redintegration in students with MBID starts at the same onset level as in TD students; and we can determine whether developmental progress (in terms of age, cognitive capacity and vocabulary size) reflects in a similar way onto redintegration development as in TD students. As redintegration is operationalized as lexicality effect (i.e., a relative benefit for real words over pseudowords), the task condition lexicality is also included as predictor besides the group factor and the DIs, but without distinguishing between different levels of word length. For ease of interpretation, Figure 3 shows the size of the lexicality effect (the difference between real words and pseudowords) in the y-axis, whereas in the regression model lexicality is included as predictor.

Using *chronological age* as DI to predict redintegration, neither a significant intercept effect for Lexicality × Group (*p* = 0.530), nor a significant interaction of Lexicality × Group × Age (*p* = 0.617) to reflect the rate of development was found. This means that students with MBID show a similar developmental pattern to TD students regarding redintegration development in relation to chronological age. With *cognitive capacity* as DI to predict redintegration, the Lexicality × Group interaction (*p* = 0.718) showed no significant difference in intercept, and a nonsignificant interaction Lexicality × Group × COG (*p* = 0.658) indicated no difference in slopes. Thus, the development of redintegration also seems to be unimpaired in students with MBID in relation to cognitive capacity. In contrast, using *vocabulary size* as DI to predict redintegration yielded both a significant difference in intercept (Lexicality × Group; *p* = 0.024) and in slope (Lexicality × Group × VOC; *p* = 0.005). The intercept difference favors students with MBID, who show greater redintegration when vocabulary size is low. In contrast, redintegration effectiveness decreases with growing vocabulary size. This reveals an unexpected differential pattern of redintegration development. Separate analyses per group indicated that in TD students, there was no significant slope effect, *F*_(1, 100)_ = 2.062; *p* = 0.154, while for students with MBID, the negative slope effect was significant, *F*_(1, 78)_ = 8.254; *p* = 0.005. This negative slope remained significant after one possible outlier with a high vocabulary score and low redintegration in the MBID group had been excluded from the analysis, *F*_(1, 77)_ = 5.267; *p* = 0.024.

Regarding the effectiveness of redintegration in MBID, findings are mixed. On the one hand, there seems to be no general differential developmental pattern, as we found similar onsets and slopes between the groups when chronological age and cognitive capacity were used as DIs. On the other hand, the relationship between redintegration and vocabulary size is differentially inverse: The greater the vocabulary size in students with MBID, the weaker their redintegration effectiveness, even after one possible outlier had been excluded. This result is clearly unexpected and deserves further discussion.

## Discussion

### Development of Working Memory Processes in Students With MBID

The aim of this study was to determine whether students with MBID showed differential developmental patterns in three aspects of verbal WM: (a) the capacity of the PL, (b) the effectiveness of rehearsal, and (c) the effectiveness of redintegration.

We found that in relation to *chronological age*, students with MBID had a lower developmental onset in PL capacity than their TD peers, as indicated by the significant intercept difference. Thus, students with MBID have a smaller PL capacity from early on. This finding is in line with other studies in the field (Mähler and Hasselhorn, 2003; Mähler, 2007; Schuchardt et al., 2010; Poloczek et al., 2014, 2016). Concerning the growth of PL capacity, development was not slowed in rate, as indicated by the nonsignificant slope effect for the interaction with age. This means that the PL capacity of students with MBID develops at a similar rate as in TD students, but they also did not catch up with their TD peers in the range of the current sample. However, we cannot exclude the possibility that they will at a later point in their development.

When PL capacity was set in relation to *cognitive capacity*, students with MBID revealed a steeper slope than TD students, which appears counterintuitive at first glance. Such an accelerated rate has also been reported in Henry (2001) and Schuchardt et al. (2011). One possible simple explanation could be that students with MBID might catch up in their development of PL capacity when they mentally mature (but see Colom et al., 2010), while the TD group may already have reached a plateau and does not further increase beyond the given level. The findings on the relationship with cognitive capacity should be interpreted with caution, as removing children with low raw scores changed the results to nonsignificant differences. Thus, replicating studies are necessary before conclusions can be drawn.

Trajectories for PL capacity as predicted by *vocabulary size* as DI were equivalent for students with MBID and TD children, providing evidence that the development of the overall functioning of the PL in relation to LTM knowledge did not differ across groups. In both groups, therefore, PL capacity development is in line with the increasing vocabulary size.

The processes of rehearsal and redintegration were examined through the effects for word length and lexicality, respectively. When comparing the word length conditions (short vs. long words, across both lexicality conditions), we found evidence for an automatic activation of rehearsal in both groups. It should be noted, however, that this only holds for the conceptualization of rehearsal activation in Hasselhorn et al. (2000). Recent studies have challenged the role of rehearsal in producing the word length effect, and Poloczek et al. (2014) found evidence that the occurrence of a word length effect is also dependent on output delays.

We found a significant overall word length effect, as short words were more likely to be remembered than long words. Students with MBID revealed a delayed onset in rehearsal, as their advantage for short words was smaller than for the TD group. The effectiveness of rehearsal seemed to develop similarly with *chronological age*, meaning that older children showed stronger rehearsal in both groups equally, as there was no difference in the developmental rate of growth in rehearsal between the groups. This is in line with Mähler and Hasselhorn (2003) finding a delay but no deviance in adolescents with MBID regarding rehearsal, and other studies that found a developmental delay for students with MBID (Henry, 2001; Hasselhorn and Mähler, 2007; Mähler, 2007). It should be noted, however, that the word length effect can be subject to a proportional scaling artifact: as the word length effect is a relative measure of difference between short and long conditions, Jarrold et al. (2015) argue that it should be set in relation to the general performance, i.e., performance in the condition of short real words. Such an artifact cannot be dismissed in the present case and is out of scope of our current investigation.

Students with MBID appeared to be delayed in the onset of development when considering their *cognitive capacity*. While in the full sample, increasing cognitive capacity did not predict rehearsal effectiveness for the TD group, students with MBID increased on rehearsal effectiveness. Maybe, students with MBID catch up in their rehearsal development as they cognitively mature. As with PL capacity, this finding should be interpreted with caution, as children with lower scores in cognitive capacity seemed to affect the results. When they were removed from the analysis, there was neither a group difference in intercept nor in slope, suggesting that students with MBID and the TD group are equal in their developmental relation between rehearsal and cognitive capacity. Further studies should explore this effect in more depth before meaningful conclusions can be drawn.

Rehearsal effectiveness depended in neither group on the *vocabulary size*, and the patterns did not differ in onset and rate of change. This was a plausible finding that vocabulary size, intended to measure lexical LTM-knowledge of words, did not predict the phonologically-based rehearsal process.

Regarding redintegration, a general benefit for real words over pseudowords could be observed, as real words are accessible in LTM making reconstruction easier. Thus, we replicated the effect that WM word span tasks depend on LTM. Regarding *chronological age*, development onset and rate of change were similar across both groups, which means that students with MBID presented neither a delayed onset nor a slowed rate of development concerning redintegration. *Cognitive capacity* did not predict redintegration effectiveness in either group. In contrast, *vocabulary size* as developmental indicator produced differential patterns in predicting redintegration. Students with MBID presented a higher effectiveness of redintegration at the lower end of vocabulary size and, as vocabulary size increased, became less effective at redintegration. Thus, students with MBID can redintegrate less efficiently than their vocabulary size would predict.

When looking at the relationship with chronological age (and when using the traditional group-matching approach), redintegration seemed to be unimpaired in students with MBID. This can be interpreted as a point of strength, indicating that they were able to make use of LTM traces for reconstruction in a similar way to their TD peers. This was qualified, however, by the negative relationship between redintegration and vocabulary size, representing a specific differential developmental pattern for MBID, because they did not seem to benefit from a higher number of words from which they could redintegrate. One should expect that a higher number of words available in the LTM would lead to a more efficient redintegration, as redintegration is conceptualized to use existing knowledge to reconstruct the trace in the short-term memory. This was not the case in students with MBID; on the contrary, a growing vocabulary size appeared to have somewhat detrimental effects on their use of LTM knowledge for reconstructing traces in the PL. Future research will have to establish whether this poses a problem of inefficient strategy use (e.g., Klauer and Lauth, 1997), poor source monitoring (e.g., Lilienthal et al., 2015), or suboptimal organization of the mental lexicon (e.g., Vitevitch et al., 2012; Kenett et al., 2016) with a greater vocabulary size causing “confusion,” or of other processes at retrieval (e.g., Dell and O'Seaghdha, 1992).

DTs served as an analysis tool to detect developmental similarities and differences concerning the WM processes of rehearsal and redintegration. They proved particularly useful in the inclusion of developmental indicators to describe and compare the development across a wider age range and investigate the relationship with other developmental indicators (in this case, cognitive capacity and vocabulary size). We would like to emphasize three innovations: First, the differentiation of a delayed onset and slowed rate for rehearsal could not be shown by the traditional group-matching ANOVA approach. Second, DTs yielded differential developmental relations for students with MBID: Cognitive capacity did not predict rehearsal in either group when taking possible outliers into account (in the full sample of students with MBID, cognitive capacity positively predicted rehearsal), but vocabulary size negatively predicted redintegration. These detailed patterns could not be detected by the ANOVA approach either. Third, the use of a greater age range proved useful for obtaining a broader picture of development instead of comparing clusters at selected narrow age slots.

While there are several advantages to the DT approach, it should be explicitly noted that in this study, the design was cross-sectional. Therefore, “development” should not be taken literally, as we measured different individuals of a wider range of chronological age, cognitive capacity, and vocabulary size. This cross-sectional type of data is similar in the standard mental age matching approach; one important difference concerns the broader range of (mental) age, which is an explicit strength of the DT approach (Thomas et al., 2009, p. 343). A longitudinal design, which can be readily implemented in the DT framework, would be able to depict “true” development. Using correlational data on the relationship between a target task and variables taken as developmental indicators can be considered a first step requiring further validation through longitudinal designs. As Thomas et al. (2009, p. 336) point out, an “initial cross-sectional design [is ideally combined] with a longitudinal follow-up,” calling for further research.

It should be noted that the reasons why a particular developmental pattern occurs are not fully clear. DTs have been primarily used as “a descriptively more powerful empirical vocabulary” and a richer tool for the description of developmental pathways (Thomas et al., 2009, p. 355), which can even be understood as a “theory-neutral marker of atypicality” (Thomas et al., 2009, p. 340). A more general view on the underlying neuroconstructivist framework can be found in Karmiloff-Smith (1998), which provides a general theoretical context but does not make specific assumptions about which factors might be the underlying causes of different developmental patterns.

### Limitations

This study is subject to methodological and theoretical limitations. The first limitation concerns the coverage of age range. Thomas et al. (2009) recommend that the TD group should cover the whole age range from the youngest mental age to the oldest chronological age. The TD group in our sample only ranged up to the age of 13;5 years (instead of 17;1 years in the group of students with MBID). Therefore, the design may be unable to detect a possible plateau or other change in developmental rate in the TD group. While this might limit the explanatory power for the range of the older students, the general findings were not affected: The intercepts are compared at the onset of development, i.e., at the youngest age measured in the MBID group, where enough data are provided. This is similarly true for the interpretation of the comparison of slopes: The more crucial comparison is at the earlier level of development, which is provided by the younger students in the TD group. It is useful to cover a wider age range in the MBID group, as it may require a longer time period for development to be detectable. This limitation is only relevant when chronological age is used as predictor. For the DIs cognitive capacity and vocabulary size, a subsample of the TD group in the sensitive range of the measures was used for comparison, thus spanning the whole range of the MBID group.

Second, methodological considerations must be taken into account. As students with MBID were recruited solely from special educational needs schools, findings are limited to this particular population and should not be generalized to a broader MBID population. Measurement invariance across the groups may not have been given in the different tasks, possibly affecting findings. Also, floor effects in long pseudowords, especially for (mentally) younger individual students of either group, may negatively impact reliability.

Third, besides word length and lexicality, many more influences have been shown to influence performance in verbal WM tasks. This poses a concern for the validity of the word length effect as a theoretically sound indicator of a phonologically, time-based rehearsal process. In particular, Jalbert et al. (2011a,b) showed that in all studies discovering an effect for word length, test items were confounded with neighborhood size. Neighborhood size is the number of “word[s] of the same length as the target that differs by only one letter.” (Jalbert et al., 2011b, p. 340). According to Roodenrys (2009), the neighborhood size effect can be explained in terms of a redintegration process (Clarkson et al., 2017; Derraugh et al., 2017). When experimentally controlling for neighborhood size, Jalbert et al. (2011b) succeeded in removing or even inversing the word length effect. This issue should be considered in future studies to construct stimuli to avoid confounding neighborhood size with word length.

### Implications

In this study, we investigated the capacity of the phonological loop and the effectiveness of two processes within the PL, rehearsal and redintegration, in students with MBID. In order to better guide and offer adequate instruction to these children with severe academic challenges, it is necessary to understand what cognitive processes they can rely on and in which domains they have particular difficulties. This knowledge may be used to adapt instruction, e.g., by making instructions shorter so that they do not depend as heavily on rehearsal. As theoretical models on the etiology of MBID do not specify which cognitive processes might be impaired, this study marks an attempt to shed some light on the availability of verbal working memory capacity and processes. Using developmental trajectories as analytical strategy, we showed that children with MBID seemed to have a delayed onset of PL capacity and rehearsal effectiveness. The rate of growth does not appear negatively affected, suggesting that the deficit at least does not seem to increase. Redintegration was investigated for the first time in this population, and the finding that it seems intact can be interpreted as a point of strength, promising an entry for possibly successful intervention strategies. However, the differential finding regarding the relationship between redintegration and vocabulary size might indicate a possible difference in cognitive structure regarding the ability to use LTM information, but calls for further replicating research.

## Data Availability Statement

The raw data supporting the conclusions of this manuscript will be made available by the authors, without undue reservation, to any qualified researcher.

## Author Contributions

GB and MG developed the theory and conceptualized and designed the study. GB and BE planned and conducted data collection and preparation. GB performed the statistical analysis and wrote the first draft of the manuscript. All authors contributed to manuscript revisions, read and approved the submitted version.

### Conflict of Interest Statement

The authors declare that the research was conducted in the absence of any commercial or financial relationships that could be construed as a potential conflict of interest.
