# Supplementary material for: Verbal Working Memory Processes in Students With Mild and Borderline Intellectual Disabilities: Differential Developmental Trajectories for Rehearsal and Redintegration
Source: Front Psychol. 2019 Jan 9;9:2581. doi: 10.3389/fpsyg.2018.02581 (PMC6345193; doi:10.3389/fpsyg.2018.02581)
Supplement: Supplementary file 1 [file Table_1.pdf]

APPENDIX Table A-1

List of word-span stimuli per condition

real words		pseudowords	
Short	long	short	long
Ball (ball)	Briefkasten (postbox)	Bork	Bestrugeln
Baum (tree)	Eisenbahn (railway)	Deil	Franulich
Eis (ice-cream)	Erdbeere (strawberry)	Fen	Karflumen[a]
Fisch (fish)	Fernseher (TV)	Grul[b]	Külinge
Haus (house)	Kneifzange (pliers)	Laum	Laujossung
Pilz (mushroom)	Lichtschalter (light switch)	Lurf[b]	Mindinnen
Schuh (shoe)	Luftballon (balloon)	Natt	Reseubelt
Stern (star)	Schaukelpferd (rockinghorse)	Sim	Schleibunder
Topf (pot)	Zahnbürste (toothbrush)	Tirk	Wuralten[a]

Note. Stimuli for the real word were taken from the German Working Memory Test Battery (AGTB 5-12, Hasselhorn et al. 2012); for each length seven stimuli for pseudowords were taken from Hasselhorn et al. (2010), adding two words for each list to match the number of words per word-pool.

[a] two additional three-syllable nonwords were added from AGTB 5-12, nonword-repetition task.

[b] two additional one-syllable nonwords were added.

APPENDIX Table A-2

Overview of developmental trajectory models for three dependent variables (DV), as predicted by chronological age (CA), cognitive capacity (COG), and vocabulary size (VOC)

DV	Group	Parameter	Developmental Indicator: CA	COG	VOC
PL capacity	overall	Intercept[a]	$F(1, 203) = 16.48$; $p < .001$; $\eta^2 = .075$	$F(1, 178) = 4.32$; $p = .039$; $\eta^2 = .024$	$F(1, 178) = 0.95$; $p = .331$; $\eta^2 = .005$
		Slope[a]	$F(1, 203) = 3.36$; $p = .068$; $\eta^2 = .016$	$F(1, 178) = 4.63$; $p = .033$; $\eta^2 = .025$	$F(1, 178) = 0.28$; $p = .595$; $\eta^2 = .002$
	MBID	Intercept[b]	$F(1, 85) = 355.87$; $p < .001$; $\eta^2 = .798$	$F(1, 78) = 216.06$; $p < .001$; $\eta^2 = .735$	$F(1, 78) = 374.19$; $p < .001$; $\eta^2 = .828$
		Slope[b]	$F(1, 85) = 32.96$; $p < .001$; $\eta^2 = .279$	$F(1, 78) = 46.41$; $p < .001$; $\eta^2 = .373$	$F(1, 78) = 21.02$; $p < .001$; $\eta^2 = .212$
	TD	Intercept[b]	$F(1, 118) = 2511.2$; $p < .001$; $\eta^2 = .955$	$F(1, 100) = 291.99$; $p < .001$; $\eta^2 = .745$	$F(1, 100) = 319.03$; $p < .001$; $\eta^2 = .761$
		Slope[b]	$F(1, 118) = 54.57$; $p < .001$; $\eta^2 = .279$	$F(1, 100) = 14.48$; $p < .001$; $\eta^2 = .126$	$F(1, 100) = 29.08$; $p < .001$; $\eta^2 = .225$
Rehearsal	overall	Intercept[a]	$F(1, 203) = 5.39$; $p = .021$; $\eta^2 = .026$	$F(1, 178) = 7.00$; $p = .009$; $\eta^2 = .038$	$F(1, 178) = 1.57$; $p = .212$; $\eta^2 = .009$
		Slope[a]	$F(1, 203) = 1.58$; $p = .210$; $\eta^2 = .008$	$F(1, 178) = 5.94$; $p = .016$; $\eta^2 = .032$	$F(1, 178) = 1.72$; $p = .192$; $\eta^2 = .010$
	MBID	Intercept[c]	$F(1, 85) = 26.53$; $p < .001$; $\eta^2 = .238$	$F(1, 78) = 10.50$; $p = .002$; $\eta^2 = .119$	$F(1, 78) = 56.72$; $p < .001$; $\eta^2 = .421$
		Slope[c]	$F(1, 85) = 6.99$; $p = .010$; $\eta^2 = .076$	$F(1, 78) = 11.62$; $p = .001$; $\eta^2 = .130$	$F(1, 78) = 7.55$; $p = .007$; $\eta^2 = .088$
	TD	Intercept[c]	$F(1, 118) = 351.63$; $p < .001$; $\eta^2 = .749$	$F(1, 100) = 50.22$; $p < .001$; $\eta^2 = .334$	$F(1, 100) = 88.65$; $p < .001$; $\eta^2 = .470$
		Slope[c]	$F(1, 118) = 15.70$; $p < .001$; $\eta^2 = .117$	$F(1, 100) = 0.003$; $p = 0.96$; $\eta^2 = .000$	$F(1, 100) = 0.93$; $p = .337$; $\eta^2 = .009$
Redintegration	overall	Intercept[a]	$F(1, 203) = 0.40$; $p = .530$; $\eta^2 = .002$	$F(1, 178) = 0.13$; $p = .718$; $\eta^2 = .001$	$F(1, 178) = 5.19$; $p = .024$; $\eta^2 = .028$
		Slope[a]	$F(1, 203) = 0.25$; $p = .617$; $\eta^2 = .001$	$F(1, 178) = 0.20$; $p = .658$; $\eta^2 = .001$	$F(1, 178) = 8.21$; $p = .005$; $\eta^2 = .044$
	MBID	Intercept[c]	$F(1, 85) = 66.06$; $p < .001$; $\eta^2 = .437$	$F(1, 78) = 30.99$; $p < .001$; $\eta^2 = .284$	$F(1, 78) = 132.71$; $p < .001$; $\eta^2 = .63$
		Slope[c]	$F(1, 85) = 1.631$; $p = .205$; $\eta^2 = .019$	$F(1, 78) = 0.17$; $p = .678$; $\eta^2 = .002$	$F(1, 78) = 8.25$; $p = .005$; $\eta^2 = .096$
	TD	Intercept[c]	$F(1, 118) = 212.29$; $p < .001$; $\eta^2 = .643$	$F(1, 100) = 17.69$; $p < .001$; $\eta^2 = .150$	$F(1, 78) = 37.54$; $p < .001$; $\eta^2 = .273$
		Slope[c]	$F(1, 118) = 0.08$; $p = .776$; $\eta^2 = .001$	$F(1, 100) = 0.97$; $p = .328$; $\eta^2 = .010$	$F(1, 100) = 2.06$; $p = .154$; $\eta^2 = .020$

Note. The three dependent variables (DV) are: capacity of the phonological loop (PL) measured by the mean performance across all four task conditions; rehearsal measured by the magnitude of the word-length effect, i.e. including length as factor in the model;

redintegration measured by the magnitude of the lexicality effect, i.e. including lexicality as factor in the model. For each DV, an overall model is estimated to compare intercept and slope between the groups; additional models are estimated separately per group to establish if the effects differ significantly from zero in each group. Intercept denotes a level difference at the youngest age measured in the MBID group. Slope denotes a difference in gradients between the groups. Three developmental indicators (DI) are used as predictors: chronological age measured in years and months, cognitive capacity (COG) measured in CFT 1-R raw scores, and vocabulary size (VOC) measured in WWT 6-10 raw scores.
[a] In the overall-models, parameters refer to differences between the groups.
[b] In the group models for PL-capacity, parameters refer to differences from zero.
[c] In the group models for rehearsal and redintegration, parameters refer to differences between the respective conditions (i.e. length and lexicality) within each group.
